# Fueling cardiac myocyte proliferation

**DOI:** 10.20517/jca.2023.47

**Published:** 2023-12-31

**Authors:** Manisha Deogharia, Priyatansh Gurha

**Affiliations:** Center for Cardiovascular Genetics, Institute of Molecular Medicine and Department of Medicine, University of Texas Health Sciences Center at Houston, Houston, TX 77030, USA.

## Abstract

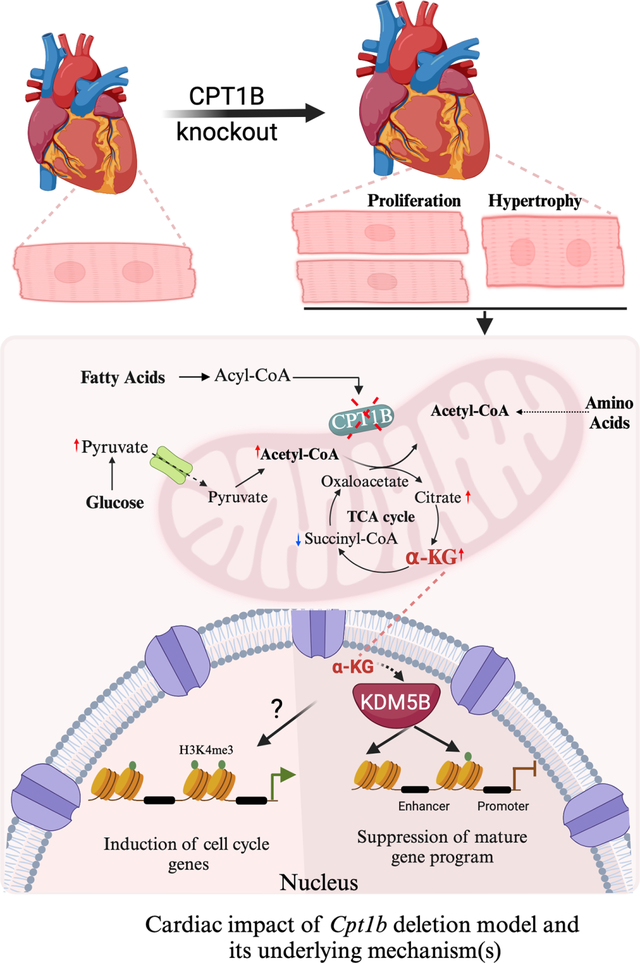

Regeneration is a biological process that is activated in response to various injuries, demonstrating the ability to restore lost components to their functional state. Therefore, it is not surprising that the concept of regeneration has fascinated humanity for centuries. In the ancient Greek myth of Prometheus, who faced punishment from Zeus for giving fire to mankind, Prometheus endured a punishment where an eagle would devour his liver every day. However, his liver would miraculously regenerate, resulting in an eternal cycle of torment and retribution for Prometheus.

From an evolutionary perspective, the ability to regenerate is widely distributed across phylogenetic lineages. For instance, hydra and planaria possess the ability to regenerate their entire bodies throughout their lives. On the other hand, lower vertebrates such as Xenopus, axolotl, and zebrafish can regrow lost limbs, tails, fins, or hearts. However, the ability of mammals to regenerate is typically limited to the early stages of development and is diminished as they mature^[1]^. Cell proliferation is a crucial process in regenerating tissue, and to support the energetically demanding process of proliferation, cellular metabolism undergoes significant changes to ensure a sufficient supply of essential nutrients. Likewise, the majority of proliferating cells utilize aerobic glycolysis for growth and proliferation.

In the heart, the ability of cardiac myocytes (CM) to proliferate decreases gradually after birth and becomes almost non-existent weeks after birth. Adult myocytes undergo terminal differentiation and lose their ability to proliferate. This aligns with the increase in oxygen availability that occurs after birth and a shift in the energy metabolism of CMs from carbohydrates like glucose and lactate to oxidative phosphorylation (OXPHOS)^[2]^. Although recent studies have highlighted several signaling pathways that can induce or facilitate the proliferation of adult CMs, the causal relationship between the progressive change in energy metabolism and the transition from quiescent to proliferative CMs remains uncertain. Therefore, investigation of the regulatory significance of different metabolic pathways and specific metabolic substrates in cardiac myocyte proliferation and heart regeneration is currently an active and promising field of research.

A recent article by Li *et al*. sheds light on the role of metabolism in cardiac myocyte proliferation and heart regeneration^[3]^. The authors reported that the expression pattern of the muscle-specific carnitine palmitoyl transferase 1b (CPT1B) is inversely correlated with CM proliferation, with *Cpt1b* being highly expressed in the adult heart while being negligible in the embryonic and neonatal heart. The CPT1B enzyme plays a crucial role in the adult heart by facilitating the oxidation of long-chain fatty acids (LCFAs) to produce ATP^[4]^. In order to study the impact of *Cpt1b*-mediated fatty acid oxidation (FAO) on CM proliferation, the authors genetically deleted this gene using two mouse models. The first model employed traditional *Myh6-Cre* mice to delete *Cpt1b* in CM during the embryonic stage. The second model utilized CM specific tamoxifen-inducible *Myh6*^MerCreMer^ mice to specifically target the *Cpt1b* gene in adult cardiac myocytes. Among the more intriguing aspects of this study, in both these models, deletion of *Cpt1b* led to an increase in the expression of the cell cycle markers phospho-Histone 3 (pH3) and Ki67, cytokinesis marker aurora kinase B, and an increase in the number of cardiac myocytes indicating CM proliferation. However, the observed changes were unexpectedly accompanied by an increase in CM cell size, indicating the presence of cardiac hypertrophy in these hearts as well. To identify the mechanism, the authors conducted RNA-seq analysis, which revealed that only a subset of the differentially expressed genes (DEGs) were shared between both the inducible and constitutive CPT1B deletion models. However, it was observed that some of the common DEGs are associated with either cell cycle regulation or the maturation of cardiac myocytes correlating with the observed phenotypic results.

The role of CPT1β in facilitating the entry of long-chain fatty acids (LCFA) into the mitochondria suggests that removing this protein could potentially affect the tricarboxylic acid (TCA) cycle. Therefore, the authors performed a metabolic screen and discovered that alpha keto glutarate (α-KG) exhibited the most substantial upregulation among the metabolites in the *Cpt1b* knockout CMs. Alpha-ketoglutarate (α-KG) is an important intermediate in the tricarboxylic acid (TCA) cycle and plays a critical role in cellular energy metabolism. In addition, it serves as a regulator of gene expression by controlling α-ketoglutarate-dependent dioxygenases such as the JmjC family of histone lysine demethylase (KDM), the TET family of DNA 5-methyl-cytosine hydroxylation, and RNA N6-methyladenosine (m6A) demethylation among others. Nevertheless, in the study by Li *et al*., the increase in α-KG levels in the *Cpt1b* knockout mice was associated with an overall decrease in H3K4me3 levels, leading the authors to speculate that α-KG may be the downstream effector of gene regulation^[3]^. The ChIP-Seq studies demonstrate redistribution of H3K4me3 and, most importantly, reduction in the broad H3K4me3 peaks at the promoters and gene body of CM maturation genes mainly involved in sarcomere formation. The H3K4me3 is typically linked to the activation of gene expression, either by an increase in RNAPII recruitment at promoters or by relieving RNAPII pausing and transcription elongation. Considering that KDM5 enzymes play a crucial role in regulating H3K4me3 levels and that α-KG is a recognized cofactor for these enzymes, the authors propose that CM proliferation is facilitated by these enzymes. Remarkably, the over-expression of KDM5B in neonatal CM exhibited increased cell proliferation markers, which was subsequently exacerbated by α-KG treatment. Similarly, the inhibition of KDM5 resulted in decreased cell proliferation induced by α-KG treatment in neonatal CM. The authors thus conclude that CM proliferation is sustained by elevated levels of α-KG in newborn hearts with reduced FAO and high KDM5 levels.

Several aspects of the present study raise further questions and open potential areas for further investigation.

1. The initial phenotypic observation indicates that the reduction in CPT1B leads to an increase in both the number of cardiac myocytes (CM) and the size of individual cardiac myocytes, i.e., hypertrophy. These two divergent phenotypes are likely caused by two distinct sets of cellular mechanisms. This prompts a critical inquiry about whether deletion of *Cpt1b* plays a dichotomous role in cellular metabolism based on the development stage of CM. Likewise, it will be interesting to identify the cell lineages of CMs that are more amenable to proliferation vs hypertrophic responses.
2. Previous studies of CPT1B deletion in mice showed an increase in heart weight: a fourfold increase in the body ratio as observed by Li *et al*., but the study also showed cardiac dysfunction^[3]^. This difference can be attributed to the non-cell autonomous effects leading to lipotoxicity^[5]^ as proposed by Li *et al*.^[3]^. Furthermore, in another study by Ghosh *et al*.^[6]^, skeletal muscle-specific deletion of CPT1B resulted in reduced α-KG, contrary to a substantial increase in α-KG as seen by Li *et al*.^[3]^. Therefore, to unequivocally rule out any deleterious effects in CM specific *Cpt1b* knockout mice, a long-term cardiac function and survival analysis is warranted.
3. Recent studies have documented the role of the KDM5 family of proteins in the maturation of cardiac myocytes^[7]^. It was shown that the levels of KDM5 gradually decrease during the maturation of CM and are negligible in the mature adult CM. In these circumstances, the present findings by Li *et al*. may be at odds with the role of KDM5 in adult CM proliferation^[3]^. A probable explanation for this paradox may be that *Cpt1b* deletion results in KDM5B re-expression in adult CMs and thereby its activity. However, the RNA levels of *Kdm5b* remained unaltered in this current study by Li *et al*.^[3]^. It is yet unknown if post-transcriptional changes can account for KDM5B protein expression in CM. Alternatively, it is also plausible that the elevated α-KG levels, as observed by Li *et al*., in the *Cpt1b* knockout CMs could promote residual KDM5B activity^[3]^.
4. While the study by Li *et al*. provides some evidence of the potential role of KDM5B in regulating the immature gene program (mostly sarcomeric genes), the precise mechanism by which KDM5B induces the expression of cell cycle remains unknown^[3]^. Furthermore, the current study did not investigate the role of other KDM5 family of proteins that are also dependent on α-KG and their contribution, if any, in the CMs. Likewise, the context-dependent role of histone modifications should also be taken into consideration. For example, several studies have shown the induction of KDM5A and B in the context of heart failure, suggesting a pathogenic role for these proteins likely through the activation of the fetal gene program.
5. Given the many pleiotropic roles of α-KG, it is conceivable that the rise in α-KG levels could potentially result in the activation of TET enzymes^[8–10]^. TET protein has been shown to play a significant role in the proliferation of cardiac myocytes and the expression of^[9]^ cardiac genes and thereby may contribute to CM proliferation and/or other phenotypes in conjunction with, or independent of KDM5s in the *Cpt1b* knockout CMs.
6. Finally, the study by Li *et al.* provides some evidence of cardiac repair mediated by *Cpt1b* in the context of Ischemic reperfusion^[3]^. Given that the process of optimal regenerating myocardium involves CM proliferation, electromechanical coupling, angiogenesis and resolution of fibrosis, and favorable immune system microenvironment, further studies will be required to establish the role of metabolic changes in the bona fide regeneration of the damaged and thereby cardiac regeneration.

Nevertheless, the current study by Li *et al*. highlights the importance of metabolism in regulating cardiac gene expression and underscores its significant role in cardiac myocyte proliferation^[3]^. The involvement of metabolites in regulating genes related to the heart offers innovative and promising opportunities for further “fueling cardiac regeneration”.
